# Supplementary material for: Vesicular glutamate transporters are H+-anion exchangers that operate at variable stoichiometry
Source: Nat Commun. 2023 May 11;14:2723. doi: 10.1038/s41467-023-38340-9 (PMC10175566; doi:10.1038/s41467-023-38340-9)
Supplement: Supplementary file 1 — Supplementary Information [file 41467_2023_38340_MOESM1_ESM.pdf]

Vesicular glutamate transporters are H^+-anion exchangers that operate at variable stoichiometry

Bettina Kolen[1,#], Bart Borghans[1,#], Daniel Kortzak[1], Victor Lugo[1], Cora Hannack[1], Raul Guzman[1], Ghanim Ullah[2], and Christoph Fahlke[1,*]

[1]Institute of Biological Information Processing, Molekular- und Zellphysiologie (IBI-1), Forschungszentrum Jülich, 52428 Jülich, Germany

[2]Department of Physics, University of South Florida, Tampa, FL 33620, USA

# contributing equally

*Correspondence: c.fahlke@fz-juelich.de

a

```
Amino-Terminus
VGLUT1_RN      -------------------M-------------EFRQEEFRKLA-------GRALGRLHR 21
VGLUT2_RN      -----MESVKQRI------L-------------APGKEGIKNFA-------GKSLGQIYR 29
VGLUT3_RN      MPFNAFDTFKEKI------L-------------KPGKEGVKNAV-------GDSLGILQR 34
                                  :               :    .:         * . *

Carboxy-Terminus
VGLUT1_RN      EEMSEEKCG----FVGHDQL---------AGSDESEMEDEVEPPGAPPAPPPSYGATHST 547
VGLUT2_RN      EETSEEKCG----FIHEDEL---------DEETGDITQNYIN-----YGTTKSYGATSQE 550
VGLUT3_RN      ENLSEEKCG----IIDQDEL---------AEETELNHEAFVSPR-----KKMSYGATTQN 554
                  .::  .     : . :             .          .         *****
```

b

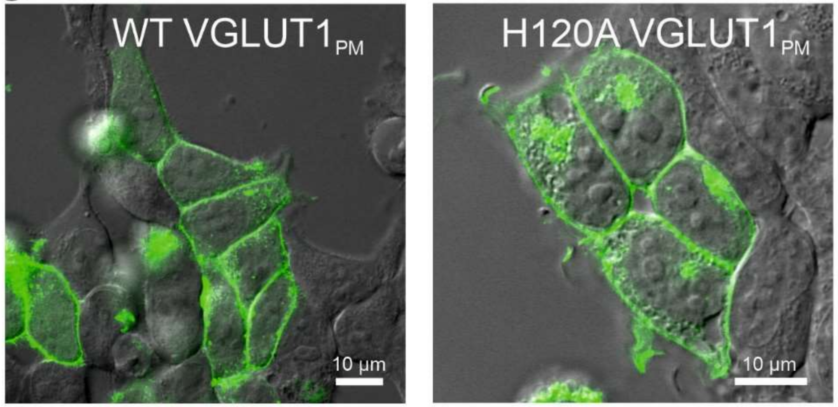

c

```
SLC17A7/vGlut1 (H.Sapiens)     [UniProtKB-Q9P2U7]   NLGVAIVSMVNNSTTHRGGHV---------------VVQKAQFSWDPETVGLIHGSFFWGYIVTQIPGGFICQKF 141
SLC17A7/vGlut1 (r.norvegicus) [UniProtKB-Q62634]   NLGVAIVSMVNNSTTHRGGHV---------------VVQKAQFNWDPETVGLIHGSFFWGYIVTQIPGGFICQKF 141
SLC17A7/vGlut1 (m.musculus)    [UniProtKB-Q3TXX4]   NLGVAIVSMVNNSTTHRGGHV---------------VVQKAQFNWDPETVGLIHGSFFWGYIVTQIPGGFICQKF 141
SLC17A6/vGlut2 (H.Sapiens)     [UniProtKB-Q9P2U8]   NLGVAIVDMVNNSTIHRGGKV---------------IKEKAKFNWDPETVGMIHGSFFWGYIITQIPGGYIASRL 149
SLC17A8/vGlut3 (H.Sapiens)     [UniProtKB-Q8NDX2]   NLGVAIVEMVNNSTVYVDGKP---------------EIQTAQFNWDPETVGLIHGSFFWGYIMTQIPGGFISNKF 154
SLC17A9/vNut (H.sapiens)       [UniProtKB-Q9BYT1]   SMPICTVSM------------------------------SQDFGWNKKEAGIVLSSFFWGYCLTQVVGGHLGDRI 87
SLC17A5/Sialin (H.Sapiens)     [UniProtKB-Q9NRA2]   NLSVALVDMVDSNTTLEDNRTSKACPEHSAPIKVHHNQTGKKYQWDAETQGWILGSFFYGYIITQIPGGYVASKI 133
SLC17A1/NPT1 (H.Sapiens)       [UniProtKB-Q14916]   CLNLTMVVMVNSTDPHGL--------PNTSTKKLLDNIKNPMYNWSPDIQGIILSSTSYGVIIIQVPVGYFSGIY 105
EAT-4a (c.elegans)             [UniProtKB-P34644]   NFGAAKTHMYKNYTDPYG------------------KVHMHEFNWTIDELSVMESSYFYGYLVTQIPAGFLAAKF 145
vGlut (D.melanogaster)         [UniProtKB-Q86P76]   NMSAAKLKGEHNGTV--------------------------FMNWTVAVESHVDSSFFWGYLVTQIPGGFIASKF 163
SLC17a7a (D.rerio)             [UniProtKB-A3QK31]   NLGVAIVSMVNDHTVYSGKKP---------------VIVAAQFTWDPETVGMIHGSFFWGYIVTQIPGGFICQKF 141
                                                     :                                   *     . : .*  :*  : *:  *..
```

Supplementary Figure 1. Molecular modifications that improve surface membrane insertion of VGLUT1 and conservation of H120 in related transporters. a Amino acid sequence alignment of VGLUT amino- and carboxy-termini. Residues E6, E7, L11, E505, E506, F520, and V511 are shown in red in the VGLUT1 sequence. **b** Confocal images of HEK293T cells transiently expressing wild type (WT) or H120A VGLUT1$_{PM}$. Representative results from four transfections with WT VGLUT1$_{PM}$ and three with H120A VGLUT1$_{PM}$. **c** Amino acid sequence alignment of SLC17 transporters from various organisms showing conservation of H120 in some, but not all, transporters.

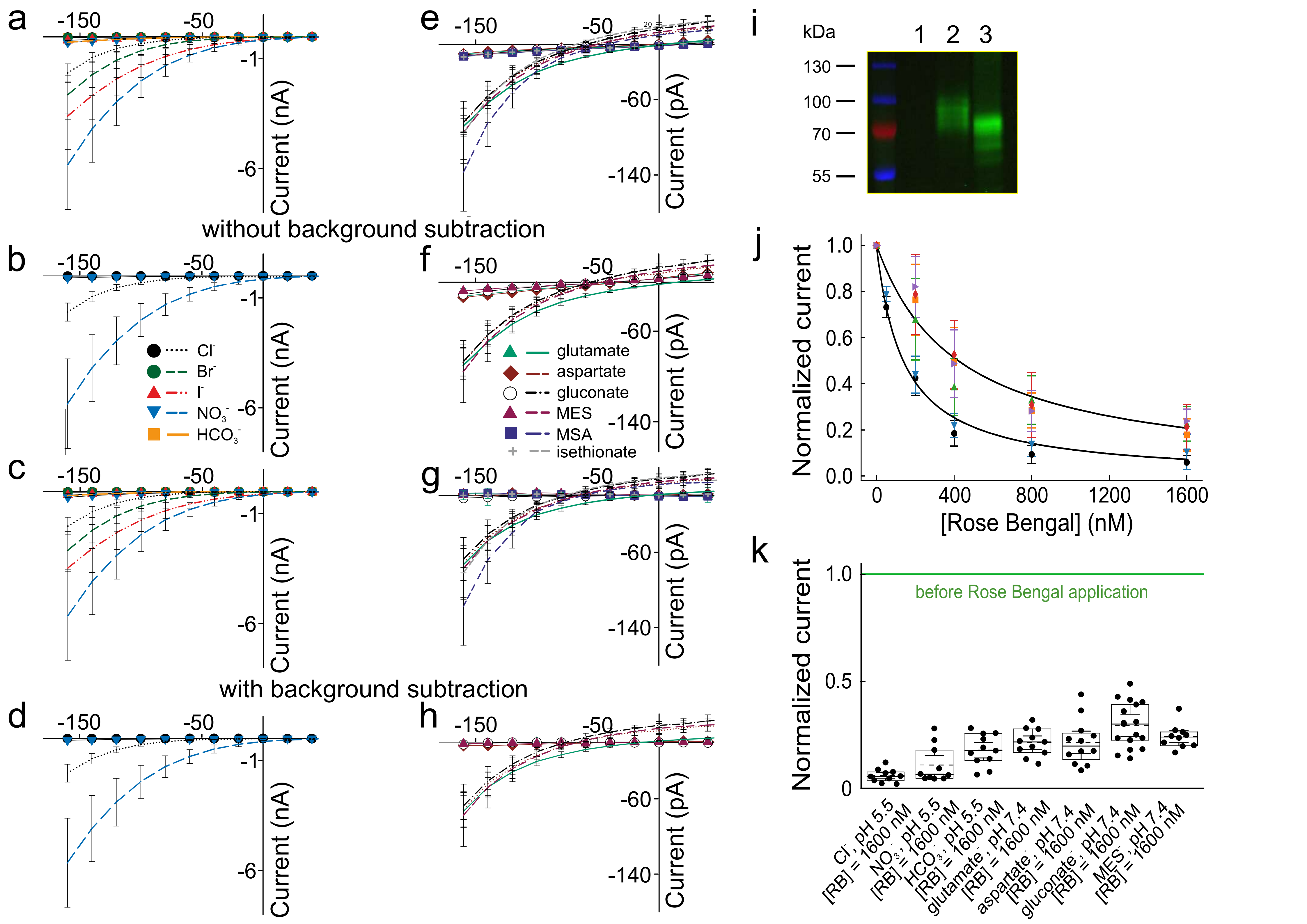
a
b
c
d
e
f
g
h
i
j
k
-150
-50
-1
-6
Current (nA)
-60
-140
Current (pA)
without background subtraction
with background subtraction
Cl-
Br-
I-
NO3-
HCO3-
glutamate
aspartate
gluconate
MES
MSA
isethionate
kDa
1
2
3
130
100
70
55
Normalized current
[Rose Bengal] (nM)
0
400
800
1200
1600
0.0
0.2
0.4
0.6
0.8
1.0
0.5
before Rose Bengal application
Cl-, pH 5.5 [RB] = 1600 nM
NO3-, pH 5.5 [RB] = 1600 nM
HCO3-, pH 5.5 [RB] = 1600 nM
glutamate-, pH 7.4 [RB] = 1600 nM
aspartate-, pH 7.4 [RB] = 1600 nM
gluconate-, pH 7.4 [RB] = 1600 nM
MES-, pH 7.4 [RB] = 1600 nM

Supplementary Figure 2. Endogenous currents only minimally contribute to background-corrected VGLUT1$_{PM}$ whole-cell currents. a, **e** Current-voltage relationships (means ± CI) from untransfected cells internally dialyzed with solutions containing indicated anions (Cl^-, n=10 cells, Br^-, n=11 cells, I^-, n=10 cells, NO_3^-, n=10 cells, HCO_3^-, n=10 cells (**a**), glutamate, n=10 cells, aspartate, n=11 cells, gluconate, n=10 cells, MES, n=13 cells, MSA, n=10 cells and isethionate, n=10 cells (**e**)) and externally perfused with 40 mM Cl^-, pH 5.5. For comparison, current amplitudes from cells expressing WT VGLUT1$_{PM}$ are given as small symbols (means ± CI, Cl^-, n=10 cells, Br^-, n=10 cells, I^-, n=10 cells, NO_3^-, n=10 cells, HCO_3^-, n=12 cells (**a**), glutamate, n=26 cells, aspartate, n=12 cells, gluconate, n=12 cells, MES, n=12 cells, MSA, n=10 cells and isethionate, n=10 cells). **b, f** Current-voltage relationships (means ± CI) from cells expressing H191K-H426K-D426Q VGLUT1$_{PM}$ and internally dialyzed with solutions containing indicated anions (Cl^-, n=10 cells, NO_3^-, n=10 cells (**b**), glutamate, n=10 cells, aspartate, n=10 cells, gluconate, n=10 cells, MES, n=10 cells, and MSA, n=10 cells (**f**)) and externally perfused with 40 mM Cl^-, pH 5.5. For comparison, current amplitudes (means ± CI) from cells expressing WT VGLUT1$_{PM}$ (same cells as shown in **a** and **e**) are given as small symbols. **c, g** Current-voltage relationships (means ± CI) from untransfected cells (same experiments as in **a** and **e)** after subtracting currents obtained at the same cell at external pH 7.4. For comparison, background subtracted current amplitudes from cells expressing WT VGLUT1$_{PM}$ are given as small symbols (means ± CI, same cells as in **a** and **e**). **d, h** Current-voltage relationships (means ± CI) from cells expressing H191K-H426K-D426Q VGLUT1$_{PM}$ (same experiments as in **b**, **f)** after subtracting currents obtained at the same cell at external pH 7.4. For comparison, background subtracted current amplitudes from cells expressing WT VGLUT1$_{PM}$ are given as small symbols (means ± CI, same data as in **c**, **g**). Significance levels for the comparison of current amplitudes at -120 mV for the data in **a** – **h** are given in Supplementary Table 1.**i** SDS-PAGE analysis of lysates from untransfected HEK293T cells (lane 1) and HEK293T cells expressing GFP-tagged WT (lane 2) or H191K-H426K-D428Q (lane 3) GLUT1$_{PM}$. Protein bands were visualized using a fluorescence gel scanner (Typhoon FLA9500, GE Healthcare, Freiburg, Germany) at 100 µm resolution, with eGFP excited at 473 nm and emissions recorded using a 530/20 bandpass filter. Representative analysis from two experiments. **j** Concentration dependence of VGLUT1$_{PM}$ anion current block by Rose Bengal. Current amplitudes measured in HEK293T cells expressing WT VGLUT1$_{PM}$ internally dialyzed with solutions containing indicated anions (means ± CI, n= 15, 12, 11, 11, 12, 10 cells for [RB] 0, 50, 200, 400, 800 and 1600) or NO_3^- (n= 12, 12, 11, 12, 10, 10 cells for [RB] 0, 50, 200, 400, 800 and 1600 mM, dashed line) or with HCO_3^- (n=15, 11, 11, 10, 11 cells for [RB] of 0, 200, 400, 800 and 1600 mM), glutamate (n=22, 10, 10, 13, 11 cells for [RB] of 0, 200,

400, 800 and 1600 mM), aspartate (n=16, 11, 11, 10, 12 cells for [RB] of 0, 200, 400, 800 and 1600 mM), or MES (n=16, 12, 12, 12, 10 cells for [RB] of 0, 200, 400, 800 and1600 mM) and externally perfused with 40 mM Cl^-, pH 5.5 were normalized to control amplitudes obtained before Rose Bengal perfusion and averaged. Lines provide fits to data obtained with internal Cl^- or NO_3^- (dashed line) or with HCO_3^- glutamate, aspartate, MES, or gluconate (solid line) with Michaelis–Menten relationships. **K** Current amplitudes of cells expressing $VGLUT1_{PM}$ in the presence of 1600 nM Rose Bengal after activation by pH 5.5. Box plots show single data as symbols, mean values dashed lines, medians as solid lines, upper and lower quartiles as box borders, and 95% CI as whiskers. Current amplitudes were normalized to amplitudes before Rose Bengal application at pH 5.5. For all anions, currents under saturating [Rose Bengal] are smaller than before Rose Bengal application at pH 5.5 (t-test: Cl^-, p = 6.9 $x10^{-15}$, NO_3^-, p = 1.2 $x10^{-10}$, HCO_3^-, p = 4.6 $x10^{-12}$, glutamate, p = 1.6 $x10^{-12}$, aspartate, p =3.4 $x10^{-11}$, gluconate, p = 6.5 $x10^{-15}$, MES, p = 1.0 $x10^{-11}$). Source data are provided in the Source Data file.

Supplementary Table 1. Significance levels of Mann–Whitney U-tests for comparisons of current amplitudes at -120 mV of cells dialyzed with solutions based on indicated anions. Cells were either transfected with WT $VGLUT1_{PM}$ (WT transfected), H191K-H426K-D426Q $VGLUT1_{PM}$ (mutant transfected) or not transfected (non-transfected)

anion	WT transfected with non-transfected cells, no background subtraction	WT transfected with non-transfected cells, background subtraction	WT transfected with mutant transfected cells, no background subtraction	WT transfected with non-transfected cells, background subtraction
Cl^-	7.86×10^{-9}	2.83×10^{-8}	1.45×10^{-5}	4.79×10^{-6}
Br^-	5.67×10^{-6}	5.67×10^{-6}		
I^-	1.08×10^{-5}	1.08×10^{-5}		
NO_3^-	1.08×10^{-5}	1.08×10^{-5}	1.83×10^{-4}	1.83×10^{-4}
HCO_3^-	5.67×10^{-6}	2.65×10^{-5}		
glutamate$^-$	7.87×10^{-9}	7.87×10^{-9}	$1.45 x10^{-5}$	1.01×10^{-5}
aspartate$^-$	1.48×10^{-6}	2.96×10^{-6}	1.50×10^{-4}	8.69×10^{-4}
gluconate$^-$	1.48×10^{-6}	1.48×10^{-6}	8.73×10^{-5}	8.73×10^{-5}
MES$^-$	1.48×10^{-6}	1.48×10^{-6}	1.83×10^{-4}	1.83×10^{-4}
MSA$^-$	5.67×10^{-6}	1.78×10^{-5}		
isethionate$^-$	1.02×10^{-6}	4.49×10^{-7}		

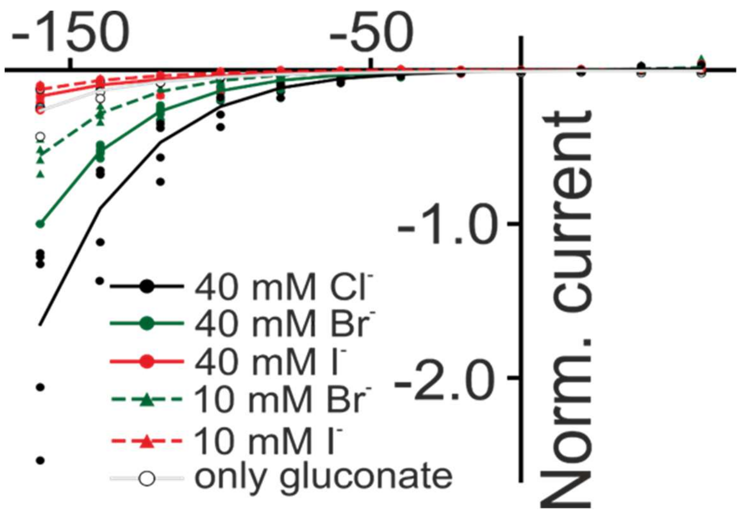

Supplementary Figure 3. VGLUT1$_{PM}$ Cl- channels are activated by Cl$^-$ and Br$^-$, but not by I$^-$. Current-voltage relationships from cells expressing WT VGLUT1P$_M$ internally dialyzed with Cl$^-$-based solutions. Cells were perfused with solutions containing 40 mM Cl$^-$ (n=5 cells), 10 mM Br$^-$ (n=4 cells), 40 mM Br$^-$ (n=9 cells)' 10 mM I$^-$ (n=4 cells), 40 mM I$^-$ (n=9 cells), or only gluconate n=4 cells). Currents were normalized to current amplitudes at -160 mV in 40 mM Br$^-$ at the same cell and given as individual data points, mean values are given as lines. Source data are provided in the Source Data file.

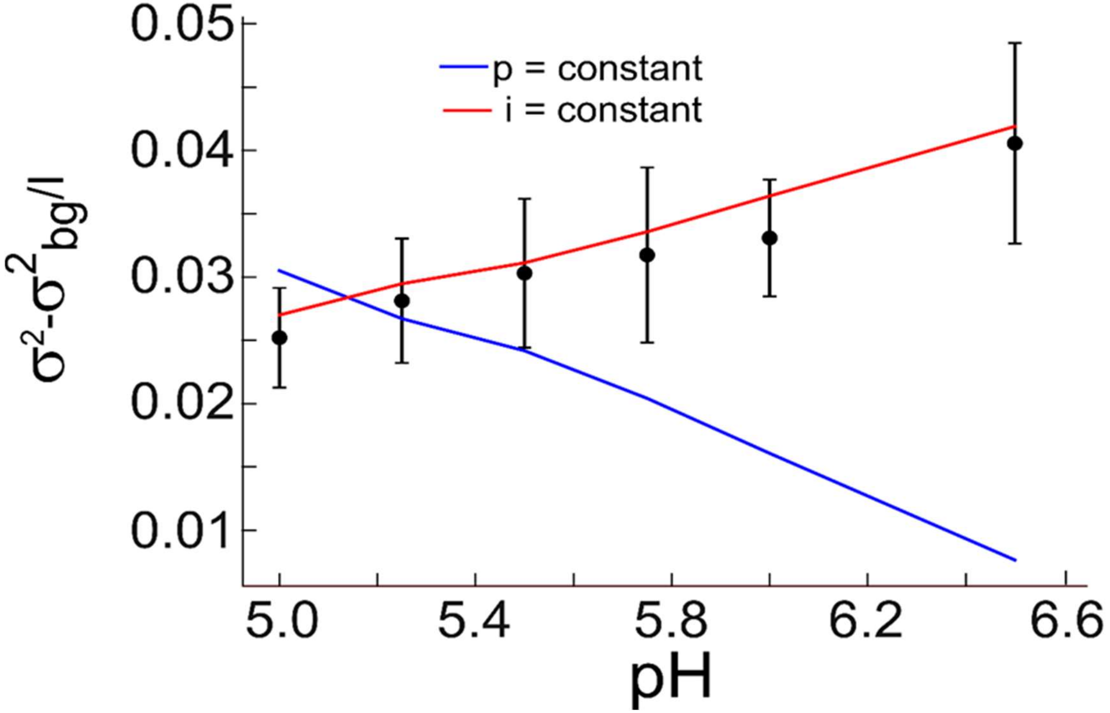

Supplementary Figure 4. Activation of VGLUT1PM anion current is based on increased open probability of VGLUT1PM anion channels. Ratios of current variances by current amplitudes ($\frac{\sigma^2-\sigma_{bg}^2}{I}$) from cells expressing WT VGLUT1PM and internally perfused with NO_3^--based solution were plotted against the external pH (means ± CI, n=11 cells). Lines are the fit assuming a variable open probability (red) or variable unitary current amplitude (blue). Source data are provided in the Source Data file.

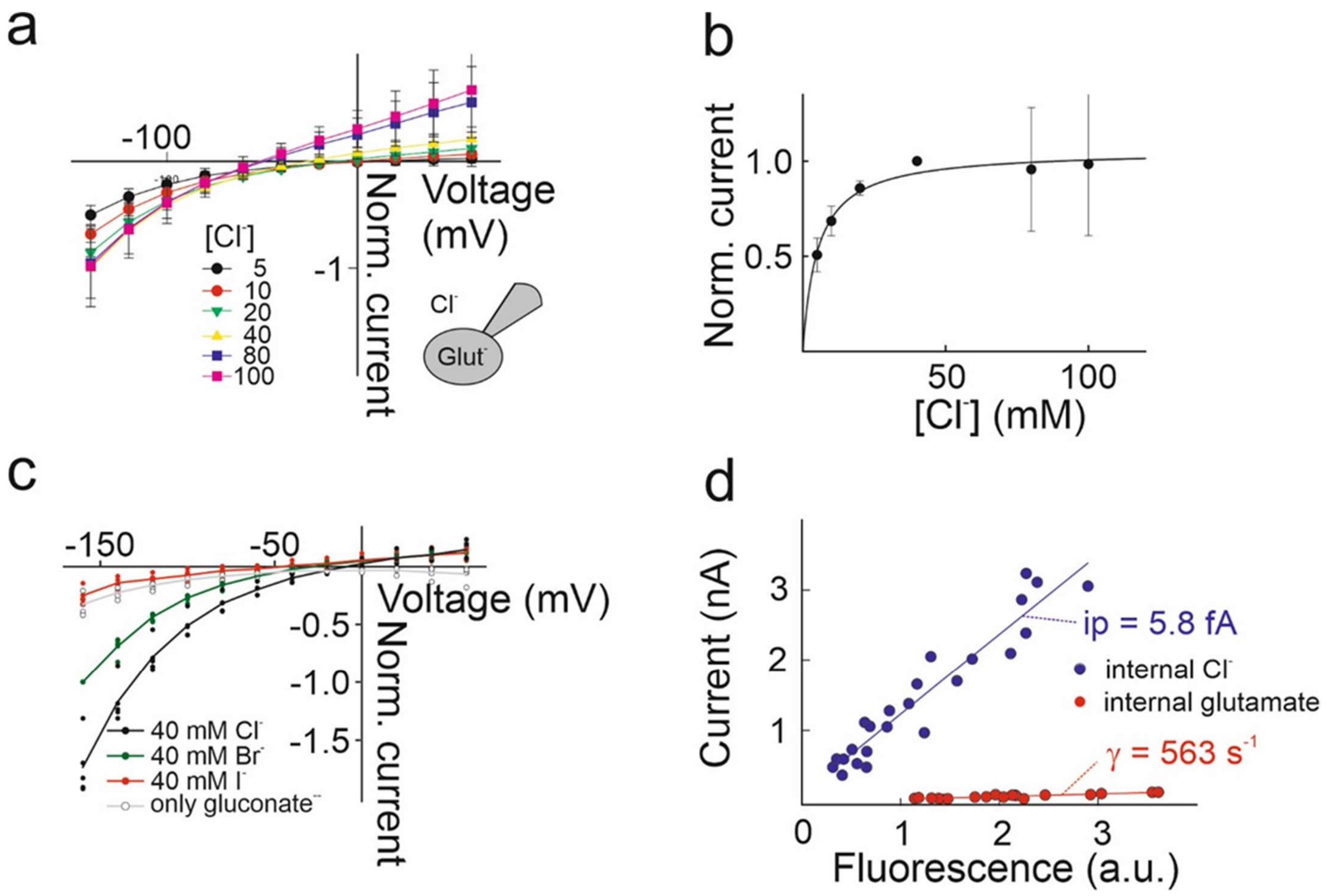

Supplementary Figure 5. Anion dependence and unitary transport rates of VGLUT1$_{PM}$ glutamate currents. a, **b** Current–voltage relationship (**a**) and dose response plots (**b**) for VGLUT1$_{PM}$ glutamate currents at rising $[Cl^-]_o$. All currents were normalized to current amplitudes at -140 mV and $[Cl^-]_o$ = 40 mM measured at the same cell (means ± CI, n =12, 12, 12, 12, 11, 10 cells). The solid line in **b** provides a fit with the Michaelis–Menten relationship. **c** Current-voltage relationships from cells expressing WT VGLUT1$_{PM}$ internally dialyzed with glutamate-based solutions. Cells were

consequently perfused with solutions containing 40 mM Br^- (n=6 cells), 40 mM Cl^- (n=5 cells), 40 mM I^- (n=5 cells), or only gluconate (n=5 cells). Currents were normalized to current amplitudes at -160 mV in 40 mM Br^- at the same cell. Symbols show individual data points, lines give mean values. **d** Plots of whole-cell current amplitudes of cells dialyzed with Cl^- (blue symbols) or with glutamate (red symbols) at -160 mV versus whole-cell fluorescence. Currents were averaged over a period of 15 ms 100 ms after the voltage step to -160 mV at pH 5.5. Differences in slope were used to calculate the unitary transport rates for H^+/glutamate exchange (563 s^{-1}) from the Cl^- transport rates obtained by noise analysis (ip = 5.8 fA). Source data are provided in the Source Data file.

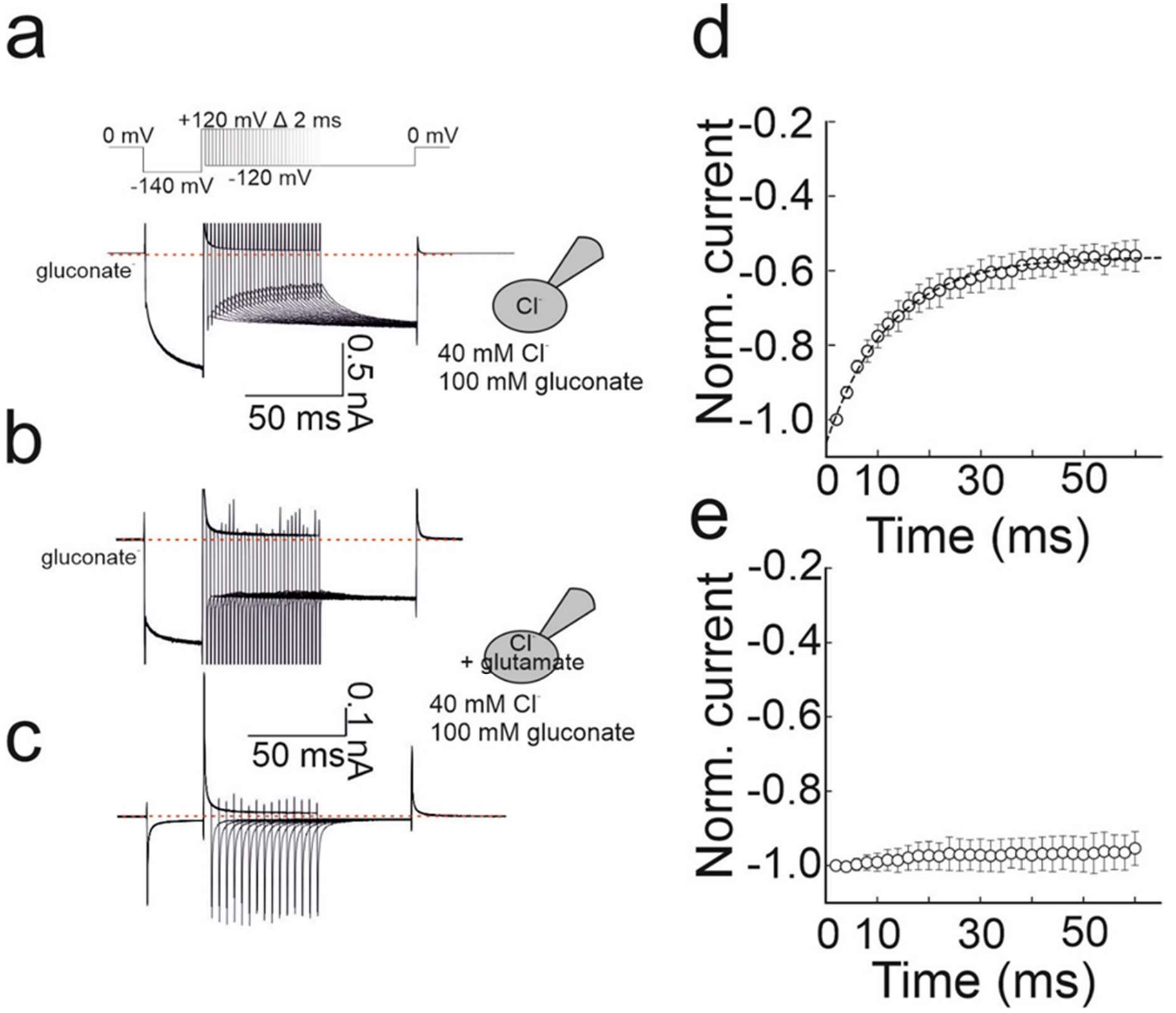

a
+120 mV Δ 2 ms
0 mV
0 mV
-140 mV
-120 mV
gluconate⁻
Cl⁻
0.5 nA
50 ms
40 mM Cl⁻
100 mM gluconate
b
gluconate⁻
Cl⁻
+ glutamate
0.1 nA
50 ms
40 mM Cl⁻
100 mM gluconate
c
d
Norm. current
-0.2
-0.4
-0.6
-0.8
-1.0
0 10 30 50
Time (ms)
e
Norm. current
-0.2
-0.4
-0.6
-0.8
-1.0
0 10 30 50
Time (ms)

Supplementary Figure 6. Glutamate impairs voltage-dependent VGLUT1$_{PM}$ anion channel closure at positive potentials. a, b Representative VGLUT1$_{PM}$ current responses to a series of voltage steps, in which a depolarizing voltage step of increasing duration is preceded and followed by fixed membrane hyperpolarization. This protocol permits quantification of the time course of VGLUT1$_{PM}$ anion channel deactivation and shows that polyatomic anions prevent complete channel deactivation at positive potentials. **c**, Averaged current responses from ten HEK293T cells expressing H191K-H426K-D428Q VGLUT1$_{PM}$ to the same pulse protocol as in **a** and **b**. **d, e** Current amplitudes determined 1 ms after the voltage step to -120 mV and normalized to the maximum value versus duration of the preceding voltage step to +120 mV (means ± CI, n=10 cells). Cells were dialyzed in an external solution containing 40 mM Cl^- and 100 mM gluconate, and currents were not background subtracted. In **a** and **d**, cells were internally dialyzed with a solution containing Cl^- as only permeable/transported anion, in **b, c** and **e** containing 40 mM Cl^- and 100 mM glutamate. Source data are provided in the Source Data file.

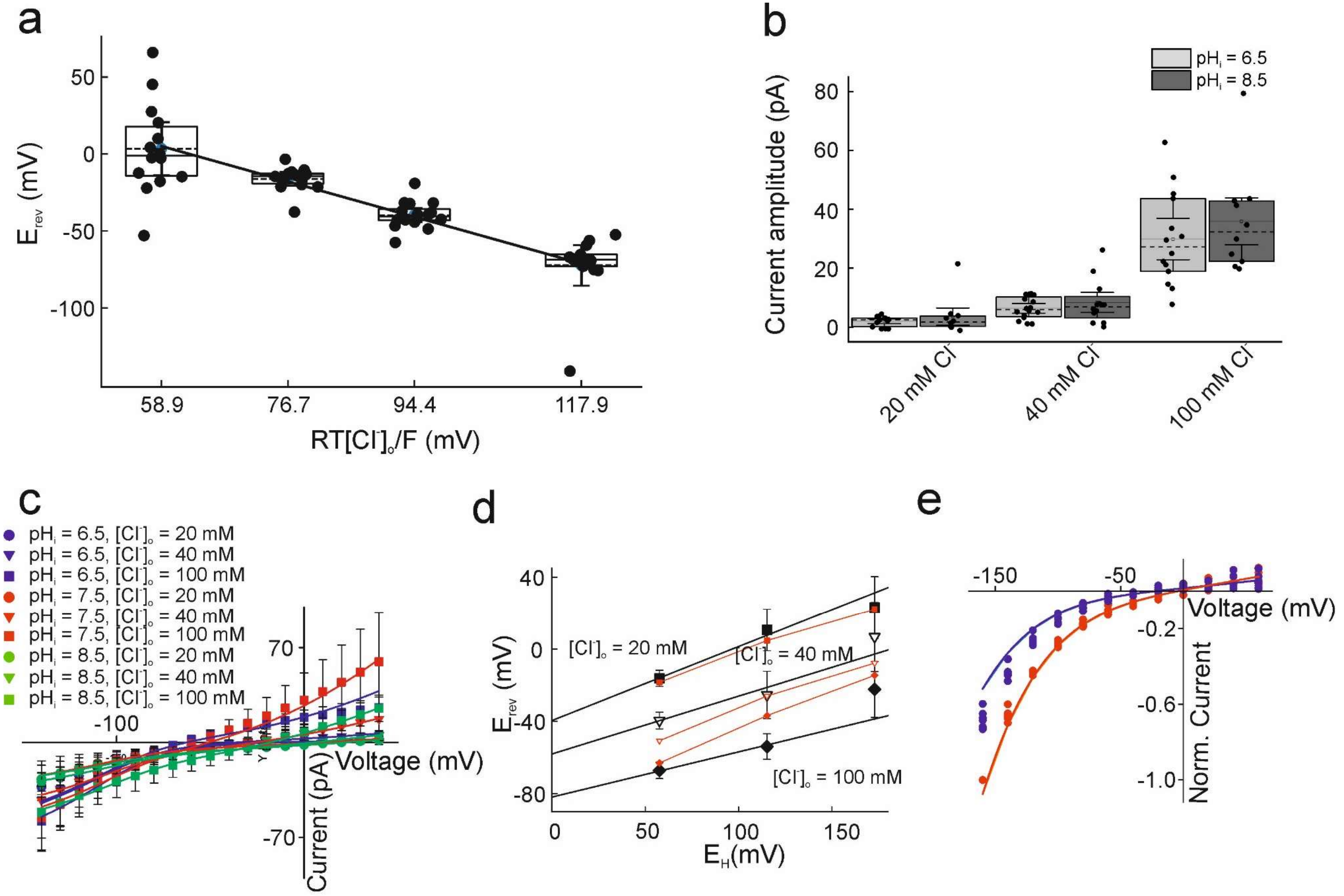
a
50
0
-50
-100
E_rev (mV)
58.9
76.7
94.4
117.9
RT[Cl-]o/F (mV)
b
pHi = 6.5
pHi = 8.5
80
60
40
20
0
Current amplitude (pA)
20 mM Cl-
40 mM Cl-
100 mM Cl-
c
pHi = 6.5, [Cl-]o = 20 mM
pHi = 6.5, [Cl-]o = 40 mM
pHi = 6.5, [Cl-]o = 100 mM
pHi = 7.5, [Cl-]o = 20 mM
pHi = 7.5, [Cl-]o = 40 mM
pHi = 7.5, [Cl-]o = 100 mM
pHi = 8.5, [Cl-]o = 20 mM
pHi = 8.5, [Cl-]o = 40 mM
pHi = 8.5, [Cl-]o = 100 mM
70
-100
Voltage (mV)
-70
Current (pA)
d
40
0
-40
-80
E_rev (mV)
[Cl-]o = 20 mM
[Cl-]o = 40 mM
[Cl-]o = 100 mM
0
50
100
150
E_H(mV)
e
-150
-50
Voltage (mV)
-0.2
-0.6
-1.0
Norm. Current

Supplementary Figure 7. VGLUT1$_{PM}$ functions as H^+-glutamate exchanger. a, Plot of VGLUT1$_{PM}$ current reversal potentials versus $\frac{RT}{F}\ln[Cl^-]_o$. In these experiments, cells were dialyzed with glutamate as only internal anion, and perfused with external mixtures of Cl^- and glutamate; $pH_o = 5.5$ and $pH_i=6.5$ (n=15, 14, 16, 14 cells). The solid lines represents a weighted fit to a linear function. The electrochemical gradient for coupled exchange of m glutamate, p Cl^- and n H^+ for is:

$$\Delta G = m\Delta G_{glutamate} - n\Delta G_H - p\Delta G_{Cl} = mRTln\frac{[Glu^-]_i}{[Glu^-]_o} - nRTln\frac{[H^+]_i}{[H^+]_o} - pRTln\frac{[Cl^-]_i}{[Cl^-]_o} - (m+n-p)FU$$

At the current reversal potential E_{rev}

$$mRTln\frac{[Glu^-]_i}{[Glu^-]_o} - nRTln\frac{[H^+]_i}{[H^+]_o} - pRTln\frac{[Cl^-]_i}{[Cl^-]_o} - (m+n-p)FE_{rev} = 0$$

$$mRTln\frac{[Glu^-]_i}{[Glu^-]_o} - nRTln\frac{[H^+]_i}{[H^+]_o} - pRTln\frac{[Cl^-]_i}{[Cl^-]_o} = (m+n-p)FE_{rev}$$

$$Erev = \frac{1}{m+\ n-p}(mE_{Glu} + nE_H - pE_{Cl})$$

$$Erev = \frac{p}{m+n-p}\frac{RT}{F}\ln[Cl^-]_o + \text{ const}$$

The slope factor of -1.29 excludes VGLUT1$_{PM}$ glutamate transport coupling with Cl^- and H^+ transport. **b,** Absolute background corrected current amplitudes at +60 mV from cells internally dialyzed with glutamate based solution at pH_i = 6.5 or 8.5 with external $[Cl^-]$ = 20 mM, 40 mM or 100 mM (n=14, 10, 16, 12, 14, 10). There are no pH-dependent differences at $[Cl^-]$ = 20 mM (Mann-Whitney U, p = 0.977), 40 mM (Mann-Whitney U, p = 0.798) or 100 mM (two-tailed t-test, p= 0.394). Box plots show single data as symbols, mean values as dashed lines, medians as solid lines, upper and lower quartiles as box borders, and 95% CI as whiskers. **c**, Current-voltage relationships (means ± CI) from cells internally dialyzed with glutamate-based solutions at pH_i = 6.5 (blue, n =14, 16, 14 cells), 7.5 (red, n =10, 12, 10 cells), 8.5 (green, n =10, 12, 10 cells). Every cell was

subsequently perfused with $[Cl^-]_o$ = 20 mM (●), 40 mM (▼) and 100 mM (■). Solid lines represent fits with sums of c Cl^- currents predicted by the Goldman-Hodgkin-Katz equation and coupled H^+-glutamate exchange currents (with 1:1 stoichiometry) multiplied with a Boltzmann term to account for rectification. **d,** Black symbols provide current reversal potentials from cells dialyzed with a glutamate-based internal solution versus E_H for three different $[Cl^-]_o$. (means ± CI, n =14, 10, 10, 16, 12, 12, 14, 10, 10 cells), red symbols are reversal potentials obtained by the fits shown in **a**. The experimental data are identical to those shown in Fig. 3d. Solid lines provide weighted linear regression to the experimentally obtained pH_i dependences of E_{rev}. **e,** Current-voltage relationships from HEK293T cells expressing WT $VGLUT1_{PM}$ (n=6, symbols show individual data points, lines mean current amplitudes). Cells were dialyzed with glutamate-based internal solutions and subsequently perfused with external solutions containing either 100 mM glutamate (red) or 100 mM gluconate (black), in addition to 40 mM Cl^-. Currents were normalized to amplitudes at -160 mV in external gluconate. Solid lines depict fits to sums of currents predicted for sums of passive Cl^- and gluconate influx and coupled glutamate-H^+ exchange multiplied with a Boltzmann term to account for rectification. Exchanging glutamate by gluconate was assumed to reduce external [glutamate] to 50 pM.

$$I\,(V) = -a\frac{VF^2[Cl^-]_o\, e^{\frac{VF}{RT}}}{RT\left(1-e^{\frac{VF}{RT}}\right)} - b\frac{VF^2[gluconate\,]_o\, e^{\frac{VF}{RT}}}{RT\left(1-e^{\frac{VF}{RT}}\right)} - c(log\frac{[H^+]_o[glu^-]_i}{[H^+]_i[glu^-]_o} - \frac{2F}{RT}V)\left(\frac{d}{1+e^{\frac{e(V-V_0)}{RT}}} + f\right)$$

Source data are provided in the Source Data file.

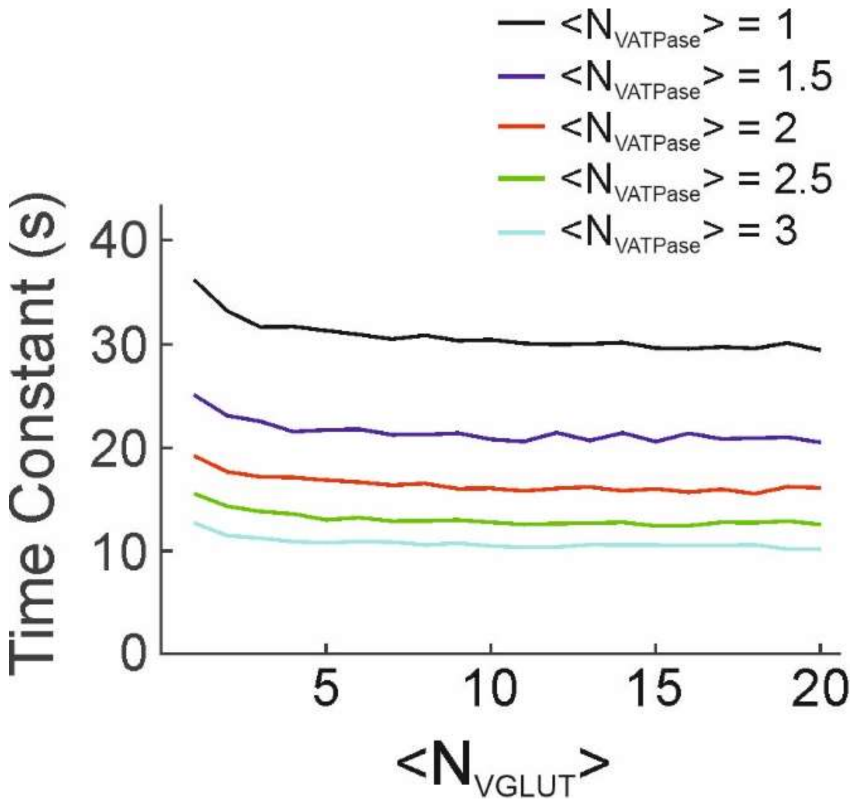

Supplementary Figure 8. The time course of glutamate accumulation in a continuum quantitative model of vesicular H^+-coupled glutamate accumulation depends on the average number of V-ATPase complexes and glutamate transporters per vesicle. Glutamate accumulation in model vesicles was simulated for various $\langle N_{VATP} \rangle$ and $\langle N_{VGLUT} \rangle$, and time courses were fitted with single exponentials. Time constants decreases as the average number of VGLUTs increases and reaches a plateau with three to five transporters per vesicle. The time scale also drops sharply upon increased average numbers of V-ATPase complexes in the vesicle.

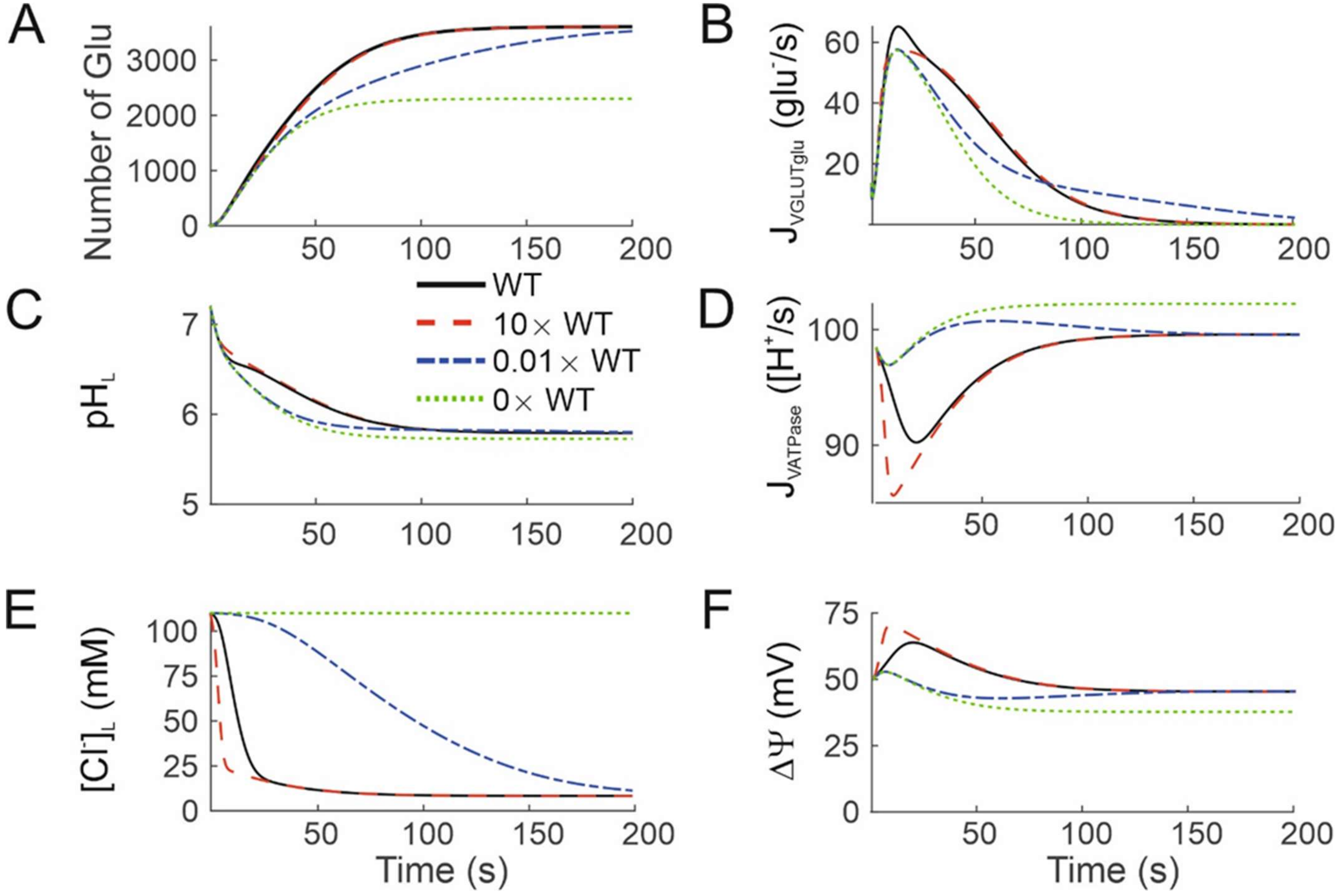

Supplementary Figure 9. Changes in VGLUT Cl⁻ conductance modify acidification, substrate accumulation, and Cl⁻ efflux in a continuum quantitative model of vesicular H⁺-coupled glutamate accumulation. a–f Predicted evolution of luminal [glutamate] (**a**), pH (**c**), [Cl⁻] (**e**), glutamate

transport rates (**b**), active proton transport rates (**d**), and Cl^- currents (**f**) by VGLUT chloride channels after increasing or decreasing the relative VGLUT Cl^- conduction.
